# Transient Platelet Elevation Following Oseltamivir Administration in Pediatric Chronic Immune Thrombocytopenia

**DOI:** 10.7759/cureus.111448

**Published:** 2026-06-24

**Authors:** Soh Yamamoto, Yu Furui, Eri Okura, Kenichi Sakamoto, Koichi Hirabayashi, Shoji Saito, Yozo Nakazawa

**Affiliations:** 1 Department of Pediatrics, School of Medicine, Shinshu University, Matsumoto, JPN

**Keywords:** chronic itp, desialylation, eltrombopag, influenza, oseltamivir

## Abstract

Oseltamivir is widely used for influenza, but its effects on platelet counts in pediatric patients with chronic immune thrombocytopenia (cITP) remain unclear. We report three adolescents with cITP (four events) who showed transient platelet elevation after oseltamivir while receiving low-dose prednisolone and eltrombopag. Platelet counts increased nine to 14 days after oseltamivir initiation (median: 11.5 days), from a median of 9.1×10^4^/μL to 26.7×10^4^/μL; two events required eltrombopag dose reduction or temporary interruption. Platelet counts subsequently returned to approximately baseline within a median of 57 days, and no thrombosis, major bleeding, or other adverse events related to thrombocytosis were observed. Although causality cannot be established and influenza itself may contribute to platelet changes, clinicians should be aware that some children with cITP may develop transient platelet elevation after oseltamivir for influenza, which can complicate eltrombopag management and warrants planned platelet monitoring.

## Introduction

Immune thrombocytopenia (ITP) is an autoimmune disorder caused by immune-driven platelet destruction and impaired platelet production [1]. Oseltamivir is commonly used to treat influenza. While several reports have described a rise in platelet counts after oseltamivir in patients with acute ITP, information on children with chronic ITP (cITP) remains limited [2,3]. We describe three pediatric patients (four events) with cITP who experienced a transient increase in platelets after oseltamivir treatment for influenza. This observation is clinically relevant because transient platelet elevation may raise concern for thrombocytosis and affect monitoring and eltrombopag dose adjustment in children with cITP.

## Case presentation

Case 1

A 15-year-old boy (immune thrombocytopenia {ITP} diagnosed at 14 years of age) had cITP treated with low-dose prednisolone and eltrombopag. He contracted influenza and received oseltamivir. His platelet count increased from 6.7×10^4^/μL before oseltamivir initiation to 34.8×10^4^/μL at 14 days after initiation, which prompted a dose reduction of eltrombopag. His platelet count decreased to 6.2×10^4^/μL at 42 days after the initiation of oseltamivir.

Case 2

A 14-year-old girl (ITP diagnosed at nine years of age) was receiving low-dose prednisolone and eltrombopag for cITP. Oseltamivir was initiated after the onset of influenza. Her platelet count increased from 6.6×10^4^/μL to 93.1×10^4^/μL at 10 days after initiation, and eltrombopag was temporarily discontinued. At her next visit (31 days after oseltamivir initiation), her platelet count had decreased to 0.6×10^4^/μL, at which time eltrombopag was restarted.

Case 3

A girl with cITP (diagnosed at 10 years of age) was maintained with low-dose prednisolone and eltrombopag. She contracted influenza twice (at 14 and 15 years of age), receiving oseltamivir on both occasions. In the first event, her platelet count increased from 11.6×10^4^/μL to 16.4×10^4^/μL at 13 days after initiation. Her ITP treatment was unchanged, and her platelet count was 8.3×10^4^/μL at 89 days after oseltamivir initiation. In the second event, her platelet count increased from 12.2×10^4^/μL to 18.7×10^4^/μL at nine days after initiation and then decreased to 8.2×10^4^/μL at 72 days, again without alterations in ITP treatment.

Taken together, we encountered four episodes of platelet increase after oseltamivir administration in three patients with cITP. All influenza episodes were managed as outpatients with fever and respiratory symptoms. Median age at ITP diagnosis was 10 years (range: nine to 14 years), with influenza onset at a median age of 14.5 (range: 14-15 years). All patients were receiving low-dose prednisolone and eltrombopag at the time of influenza diagnosis. Median platelet count increased after oseltamivir initiation from 9.1×10^4^/μL (range: 6.6-12.2×10^4^/μL) to 26.7×10^4^/μL (range: 16.4-93.1×10^4^/μL) after a median of 11.5 days (range: nine to 14 days). Two patients required a dose reduction or temporary interruption of eltrombopag due to the risk of thrombocytosis. No thrombosis, major bleeding, or other adverse events related to thrombocytosis were observed. In all cases, the increase in platelet count was transient, with counts returning to baseline within a median of 57 days (range: 31-89 days). Key clinical details are summarized in Table 1.

**Table 1:** Clinical characteristics, ITP treatment, and serial platelet counts before and after oseltamivir in three children with chronic ITP. Cases 3a and 3b indicate two separate influenza episodes in the same patient. Values in parentheses indicate days after oseltamivir initiation. Pre-oseltamivir platelet counts represent the most recent values obtained before oseltamivir initiation. Post-oseltamivir platelet counts represent the highest recorded values during routine clinical follow-up and may not reflect the true peak. PSL: prednisolone; EPAG: eltrombopag; ITP: immune thrombocytopenia

Case	Age at influenza (years)	Sex	Platelet count (×10⁴/μL)			ITP treatment		Adverse events
			Before oseltamivir	Highest recorded after oseltamivir (day)	Subsequent follow-up (day)	Before oseltamivir	Adjustment after platelet increase	
1	15	Male	6.7	34.8 (14)	6.2 (42)	PSL 1 mg, EPAG 50 mg	EPAG reduced to 37.5 mg	None
2	14	Female	6.6	93.1 (10)	0.6 (31)	PSL 2 mg, EPAG 37.5 mg	EPAG temporarily discontinued	None
3a	14	Female	11.6	16.4 (13)	8.3 (89)	PSL 1 mg every other day, EPAG 12.5 mg	No change	None
3b	15	Female	12.2	18.7 (9)	8.2 (72)	PSL 1 mg, EPAG 12.5 mg	No change	None

## Discussion

Oseltamivir and other neuraminidase inhibitors attenuate platelet desialylation, which may suppress platelet clearance through the hepatic Ashwell-Morell receptor pathway and ultimately increase platelet count. In ITP patients, especially those with anti-GPIbα antibodies, platelet desialylation is particularly enhanced and is closely related to thrombocytopenia [4]. Recent reports on cITP have described temporary platelet increases and occasional remission after oseltamivir administration, although the degree and duration of each response varied [2,3].

In addition to these reports, several recent studies have provided further clinical evidence for platelet increases after oseltamivir. Shaim et al. retrospectively evaluated 385 patients tested for influenza and reported a greater increase in platelet counts in both influenza-infected and non-infected patients who received oseltamivir, with similar increases between these two groups, suggesting that the effect may not be explained solely by influenza infection or recovery [5]. Muthiah et al. also demonstrated a significant increase in platelet counts after oseltamivir in a large database analysis of hospitalized patients, showing an average 1.14-fold (14%) rise; notably, only a minority had a positive influenza test, and the increase did not differ significantly in influenza-positive patients compared with the overall population [6]. Pediatric evidence is also emerging; Liu et al. reported three children with ITP who failed first-line therapy and responded to oral oseltamivir phosphate [7].

Our pediatric cases also exhibited varying degrees of increase, all of which were transient. Thus, although specific antiplatelet antibodies were not measured in our series, the type of autoantibody may affect the extent of platelet increase after neuraminidase inhibitor administration. Moreover, this pattern can influence the management of pediatric patients receiving thrombopoietin receptor agonists (TPO-RAs). As eltrombopag dosing is titrated based on platelet count, prescribing information recommends dose reduction or temporary interruption above specific platelet thresholds to avoid excessive platelet elevation and potential thrombotic complications. Awareness of a possible transient rise in platelet count after oseltamivir may help clinicians modify eltrombopag dosing according to platelet changes and monitor children with cITP receiving TPO-RA therapy.

This report is limited by the small number of cases and non-standardized timing of platelet measurements and, therefore, cannot prove causality. Recovery from influenza, spontaneous platelet fluctuations in cITP, and concurrent treatment with prednisolone and eltrombopag may also have influenced the observed platelet changes. However, no intensification of ITP treatment preceded the increases in platelets. In cases 1 and 2, eltrombopag was reduced or interrupted only after the elevated platelet counts were identified and may therefore have contributed to the subsequent decline. Although prior studies suggest that platelet increases after oseltamivir may occur independent of influenza infection, our observations were made in the setting of influenza, and we cannot disentangle the effects of infection and oseltamivir in this small series.

## Conclusions

In conclusion, some children with cITP may show a transient increase in platelets after oseltamivir treatment for influenza, which may require temporary adjustment of eltrombopag. These preliminary findings should be confirmed in larger studies with appropriate comparison groups.
